# Incidental Discovery of a Sigmoid Colon Gastrointestinal Stromal Tumor Mimicking Colitis: A Case Report

**DOI:** 10.7759/cureus.91194

**Published:** 2025-08-28

**Authors:** Neel Patel, Christopher Beskow, Tristan Patel, Alexander Crean, Richard Virgilio

**Affiliations:** 1 Internal Medicine, Edward Via College of Osteopathic Medicine, Auburn, USA; 2 Neurology, Edward Via College of Osteopathic Medicine, Auburn, USA; 3 General Surgery, HCA Florida Memorial Hospital, Jacksonville, USA; 4 Colon and Rectal Surgery, HCA Florida Memorial Hospital, Jacksonville, USA; 5 Clinical Affairs, Edward Via College of Osteopathic Medicine, Auburn, USA

**Keywords:** colitis associated cancer, colon resection, gastrointestinal stromal tumor (gist), sigmoid colon, unexplained abdominal pain

## Abstract

Gastrointestinal stromal tumors (GISTs) of the colon are extremely rare and often present diagnostic challenges due to overlapping features with inflammatory or other neoplastic conditions. We report the case of a 43-year-old female initially referred for evaluation of presumed colitis who was found on colonoscopy to have a sigmoid mass. Initial biopsies showed inflammation without malignancy, but subsequent imaging and pathology confirmed a GIST. After multidisciplinary evaluation, she underwent an uncomplicated robotic-assisted partial colectomy. Final pathology confirmed a 2.2 × 1.9 × 0.6 cm low-mitotic index GIST positive for CD117 and DOG-1, with no residual disease. Postoperative recovery was favorable; adjuvant management is ongoing. This case highlights the importance of maintaining a broad differential for colonic masses and demonstrates how early multidisciplinary intervention can guide optimal surgical and oncologic care, even in atypical GIST presentations.

## Introduction

Gastrointestinal stromal tumors (GISTs) are rare mesenchymal neoplasms that arise from interstitial cells of Cajal (ICCs), which primarily function as pacemaker cells for gastrointestinal motility via regulation of gastrointestinal peristalsis [1]. GISTs account for 1-3% of all gastrointestinal malignancies and most frequently occur in the stomach (60-70%) and small intestine (20-30%) [2]. Colonic GISTs are exceedingly rare, accounting for only 2.9-9.3% of all GISTs, with an estimated incidence of approximately 0.018 per 100,000 individuals [2-3]. This rarity contributes to the scarcity of large-scale studies, with most available data derived from small case series and individual case reports [3].

The clinical presentation of colonic GIST is highly variable. In a retrospective review of 58 colonic GIST cases, tumors were incidentally discovered in over half of patients through imaging, colonoscopy, or colectomy [3]. Meanwhile, symptomatic patients reported abdominal pain (12.1%) and gastrointestinal bleeding (10.3%) [3]. Anatomically, colonic GISTs are predominantly located in the left colon (52%), followed by the right (35%) and transverse colon (13%) [3]. Despite their smaller median size (1.4 cm), colonic GISTs particularly exhibit aggressive behavior, with higher mitotic indices and lower five-year cancer-specific survival rates compared to gastric or small intestinal GISTs [2].

A diagnosis of GIST is confirmed by histopathological evaluation and immunohistochemistry. Most tumors exhibit positive expression for CD117 (KIT), a transmembrane tyrosine kinase receptor, and DOG-1, which are characteristic markers of GISTs [4]. Subsequently, they also lack the expression of desmin and S-100, which are findings that support their origin from the ICCs [4]. This novel case report describes a 43-year-old female with a descending colonic GIST that initially presented as colitis. The diagnosis was ultimately confirmed through surgical resection and immunological analysis. Given the rarity and diagnostic complexity of colonic GISTs, this case contributes to the growing literature on atypical presentations and emphasizes the importance of multidisciplinary coordination in their evaluation and treatment.

## Case presentation

**Figure 1:**
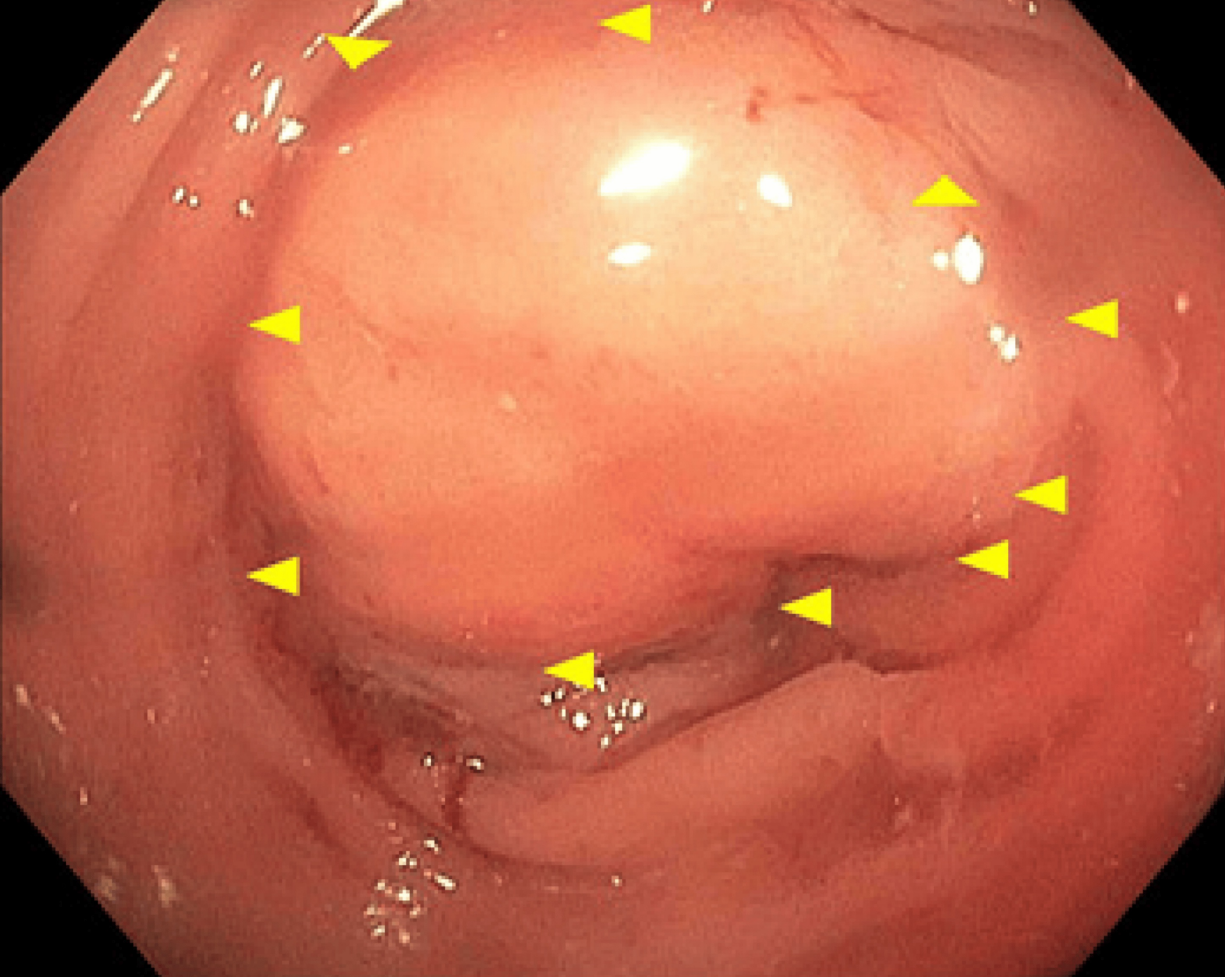
Colonoscopic image showing a partially obstructing sigmoid mass (arrow heads) located ~45 cm from anal verge

A 43-year-old female with a medical history of hypertension was referred to the Colon and Rectal Surgery Service at HCA Florida Memorial Hospital for evaluation of a suspected sigmoid colon mass. She had undergone a diagnostic colonoscopy earlier that month due to a two-week history of non-bloody diarrhea. The procedure revealed a mass located approximately 45 cm from the anal verge, in the sigmoid colon, with associated rectal inflammation as seen in Figure 1. Biopsies from both the mass and the inflamed mucosa demonstrated inflammatory changes without evidence of malignancy; pathology review was ongoing at the time of referral.

The patient denied any prior episodes of hematochezia, chronic diarrhea, or any personal or family history of colorectal or inflammatory bowel disease. She did report chronic stool evacuation difficulty. A contrast-enhanced CT scan of the abdomen and pelvis was performed, revealing no evidence of distant metastatic disease.

Subsequent pathology review confirmed the diagnosis of a GIST in the sigmoid colon. The case was presented at a multidisciplinary tumor board, and surgical resection was recommended.

The patient underwent an uncomplicated robotic-assisted partial colectomy. She tolerated the procedure well, progressed appropriately postoperatively, and was discharged in stable condition on postoperative day three.

Final histopathology identified a 2.2 × 1.9 × 0.6 cm GIST involving the descending colon, with tumor extension to the serosal surface and fewer than five mitoses per 50 high-power fields. All surgical margins were clear. Immunohistochemical analysis showed strong positivity for CD117 and DOG-1, and the Ki-67 proliferation index was <1%, consistent with a low-risk GIST. No histologic abnormalities were observed in proximal colonic segments.

At postoperative follow-up, the patient reported mild constipation managed effectively with stool softeners. She resumed a normal diet and reported no significant complaints. Further management, including consideration of adjuvant therapy, is ongoing under multidisciplinary care.

## Discussion

Despite presenting at earlier tumor (T1-T2) stages in many cases, colonic GISTs exhibit very poor cancer-specific survival (CSS) compared to GISTs in the stomach or small intestine, primarily due to their higher histologic grade and more aggressive biological behavior. Notably, the five-year CSS for colonic GISTs is approximately 71.5%, which is lower than for gastric GISTs [2]. Prognosis is most strongly correlated with tumor size, mitotic index, and symptoms at presentation [3].

The pathogenesis of GISTs is driven by mutations in receptor tyrosine kinases, particularly in the KIT and PDGFRA genes [1]. These mutations result in constitutive activation of the receptor, which subsequently triggers downstream signaling pathways that drive cellular proliferation and survival. KIT mutations, particularly those in exon 11, are the most common and exhibit aggressive behavior. Immunohistochemical staining for CD117 (KIT) and DOG-1 is central to diagnosis as nearly all GISTs express these markers robustly [4]. Immunohistochemistry also helps rule out other tumors in the differential diagnosis, since they typically lack desmin and S-100 expression, which serve as markers of smooth muscle and neural differentiation, respectively, thus aiding in the differentiation of GISTs from leiomyomas and schwannomas [5-6]. Some GISTs with epithelioid morphology instead carry PDGFRA mutations, including the imatinib-resistant exon 18 D842V variant [7]. Histologically, most colonic GISTs are of the spindle cell subtype (92%), with the remainder exhibiting epithelioid or mixed morphology [3]. Despite their small size (1.4 cm), features such as necrosis or a high mitotic index are warning signs of aggressive potential [8].

Surgical resection remains the cornerstone of curative treatment and significantly improves overall survival. Approximately 80% of patients with colonic GIST undergo surgery, most commonly via segmental colectomy [2]. In high-risk cases and rare presentations, particularly those with larger size or incomplete resection margins, adjuvant therapy with tyrosine kinase inhibitors (TKIs) such as imatinib has been shown to reduce recurrence rates [9]. However, colonic-specific data on the efficacy of TKIs are currently limited.

Recent studies highlight the evolving role of surgery in the treatment of advanced, imatinib-resistant GIST. A 2025 cohort study showed surgical intervention improved overall survival, especially in these treatment-refractory settings [10]. Novel TKIs such as avapritinib offer an option for specific resistant mutations, but brain hemorrhage has been reported as an adverse effect [11].

From a translational perspective, patient-derived xenograft models are becoming crucial in providing detailed, mutation-specific drug testing and insights into the resistance patterns for these tumors [12].

A recent retrospective series of 72 colonic GISTs (predominantly in the sigmoid colon) demonstrated significantly worse disease-free and disease-specific survival compared to gastric GISTs, with mitotic index emerging as the sole independent predictor of prognosis-findings that align with ours in underscoring poorer outcomes for colonic tumors and the prognostic importance of mitotic activity [13]. Our findings are consistent with previously reported cases in the literature, which describe sigmoid colonic GISTs as rare, often presenting with nonspecific gastrointestinal symptoms and demonstrating immunohistochemical positivity for c-KIT and DOG1; however, our case expands upon prior reports by highlighting the diagnostic value of combining colonoscopy with cross-sectional imaging to localize and characterize the lesion before surgical management.

## Conclusions

While GISTs most commonly arise in the stomach and small intestine, colonic GISTs are rare and underreported. This case highlights the diagnostic challenges and therapeutic considerations associated with the management of atypical presentations of colonic GISTs, particularly when initial biopsies are non-diagnostic. A collaborative approach encompassing comprehensive clinical assessments, imaging and the combination of histological and molecular profiling facilitates the diagnosis and management of patients.
